# Molecular and Genetic Markers for Malignant Melanoma: Implications for Prognosis and Therapy

**DOI:** 10.3390/dermatopathology12030031

**Published:** 2025-09-12

**Authors:** Lauren Fleshner, Alyssa Sayegh, Mehmet Fatih Atak, Rahim Hirani, Banu Farabi, Bijan Safai, Shoshana Marmon

**Affiliations:** 1 School of Medicine, New York Medical College, Valhalla, NY 10595, USAasayegh4@student.nymc.edu (A.S.); rhirani2@student.nymc.edu (R.H.);; 2 Dermatology Department, NYC Health + Hospital/Metropolitan, New York, NY 10029, USA; 3 Dermatology Department, NYC Health + Hospital/South Brooklyn Health, Brooklyn, NY 11235, USA

**Keywords:** melanoma, cutaneous melanoma, biomarkers, immunohistochemistry, micro-RNA, immune-checkpoint inhibitors

## Abstract

Despite therapeutic advancements, malignant melanoma remains a leading cause of skin cancer-related mortality, with incidence continuing to rise globally. Traditional prognostic tools offer important clinical guidance but fail to capture the biological heterogeneity of melanoma or reliably predict responses to emerging therapies. In this review, we summarize recent advances in prognostic and predictive molecular biomarkers reported over the past five years. We discuss immunohistochemical and tissue-based markers, circulating biomarkers, microRNAs, and gene expression profiles that enhance risk stratification and inform surveillance strategies. We also review immune-related markers that may predict response to immune-checkpoint inhibitor therapy. Lastly, we highlight investigational biomarkers—including gene signatures, epigenomic alterations, and microbiome influences—that are shaping the future landscape. Together, these advances reflect a shift toward precision oncology in melanoma, with the integration of biomarker-driven strategies offering the potential to personalize treatment and improve patient outcomes.

## 1. Introduction

Malignant melanoma remains a leading cause of cancer-related deaths, and its incidence continues to increase worldwide [1]. In 2020, there were an estimated 325,000 new cases of melanoma and 57,000 melanoma-related deaths globally; these numbers are projected to rise to 510,000 cases and 96,000 deaths by 2040 [2]. Although melanoma remains a significant public health concern, mortality rates have gradually declined since the introduction of immune-checkpoint inhibitors (ICIs) [3]. However, because ICIs are effective only in a subset of patients and carry significant toxicity and cost, the identification of biomarkers that predict treatment response is essential to guide therapy and optimize outcomes [4].

Several melanoma staging algorithms are used to determine prognosis and guide treatment decisions, with the American Joint Committee on Cancer (AJCC) tumor/node/metastasis (TNM) system and National Comprehensive Cancer Network (NCCN) guidelines among the most widely adopted [5,6]. Prognostic factors in melanoma include age, ethnicity, primary location, mitotic rate, ulceration, Breslow depth, sentinel lymph node (SLN) status, and the presence of metastasis [5,7]. While these clinicopathological variables provide important information, they do not adequately account for the underlying biological heterogeneity of melanoma or predict response to modern therapies such as immunotherapy and targeted agents [8]. In recent years, molecular and immunologic advances have led to the development and validation of assays with promising potential to improve risk stratification, better predict treatment response, and provide earlier detection of disease recurrence. This narrative review summarizes advances over the past five years in molecular and genetic biomarkers with prognostic relevance in cutaneous malignant melanoma.

A literature search of PubMed and Google Scholar was completed in May 2025. The search was limited to peer-reviewed studies published within the last five years (May 2020 to May 2025). The following search terms were used: “melanoma” AND (biomarkers OR “prognostic biomarker” OR “gene expression” OR immunohistochemistry OR “risk stratification”) AND (survival OR recurrence OR prognosis). The search was supplemented by a manual review of pertinent references. Excluded from the search were case reports, case series, editorials, commentaries, abstracts, poster presentations, and non-English publications.

## 2. Immunohistochemical and Tissue-Based Prognostic Markers

### 2.1. Preferentially Expressed Antigen in Melanoma (PRAME; Gene Symbol PRAME)

Preferentially Expressed Antigen in Melanoma (PRAME) is a tumor-associated antigen that was first identified in a patient with metastatic cutaneous melanoma and belongs to the cancer-testis antigen family [9]. It has since emerged as a potential prognostic biomarker in melanoma (Table 1), with several studies reporting that high PRAME expression (typically defined as ≥50% positive tumor cells as PRAME-positive, though individual study cutoffs may vary). Additionally, some studies use H-score systems that incorporate immunohistochemical staining intensity, which is associated with a worse prognosis and reduced overall survival (OS) [9,10,11]. In a study of 123 cases of cutaneous melanoma, Asato et al. reported that both the extent and intensity of PRAME expression were individually associated with decreased OS (*p* = 0.0267 and *p* = 0.043, respectively) [10]. PRAME has also been reported to be highly expressed in uveal melanomas and its presence correlated with an increased risk of metastasis, particularly to the liver [11]. In metastatic melanoma, PRAME expression serves as a useful marker to distinguish true lymph node metastases from benign nodal melanocytic deposits [12]. It has also shown value in acral melanoma, where it improves diagnostic accuracy by helping differentiate malignant tumors from benign acral melanocytic proliferations that can closely mimic melanoma [13].

### 2.2. Ki-67

Ki-67 is a well-established marker of cellular proliferation that has been identified in many cancer types [74]. A 2021 meta-analysis by Liu et al. evaluated the prognostic role of Ki-67 in 929 patients across 10 melanoma studies and found that its overexpression, typically defined as ≥20% proliferation index considered a “high” cutoff (most studies had cutoffs ranging from 10 to 30%, with most studies deriving these thresholds post hoc), was associated with worsened OS (HR = 2.92; 95% CI, 2.17–3.91, *p* < 0.0001) [14]. However, the authors reported no apparent correlation between Ki-67 overexpression and overall progression-free survival (PFS) (HR = 0.999; 95% CI, 0.958–1.041; *p* = 0.958; I^2^ = 21.80%; *p* = 0.258) or relapse-free survival (RFS) (HR = 1.14; 95% CI, 0.42–3.11; *p* = 0.993; I^2^ = 85.00%, *p* = 0.01) [14]. Similarly, Asato et al. found that Ki-67 expression was significantly associated with worse OS (*p* = 0.005) and sentinel lymph node (SLN) status (*p* = 0.0403) [10]. Additionally, Du et al. reported that patients with acral melanoma in the Ki-67 high expression group had significantly shorter median melanoma-specific survival than those in the low Ki-67 expression group (*p* < 0.001) [15].

### 2.3. Activating Molecule in Beclin1-Regulated Autophagy (AMBLor)

Activating molecule in Beclin1-regulated autophagy (*AMBRA1* gene), in combination with loricrin (termed “AMBLor” together), has been recently validated as a prognostic biomarker of nonulcerated AJCC stage I/II melanomas [16]. AMBLor is a combined epidermal immunohistochemical marker panel that assesses loss of AMBRA1, an autophagy-regulatory protein, with loricrin, a marker of terminal keratinocyte differentiation. In a large multivariate study by Ewen et al., absence of AMBLor expression in nonulcerated AJCC stage I–II melanoma was associated with an increased risk of recurrence (HR = 3.49; *p* = 0.007). Also, AMBLor positivity was strongly associated with favorable outcomes, with a negative predictive value of 96.5% for recurrence [16]. These findings suggest that AMBLor may serve as a valuable tool for identifying patients with early-stage melanoma who are at low risk of recurrence.

### 2.4. Y-Box Binding Protein 1 (YB-1)

Y-box binding protein (YB-1) is a multifunctional transcription and translational factor involved in cell proliferation, stress response, and oncogenesis [17]. It serves as a marker of epithelial-to-mesenchymal transition, a process that, despite melanoma’s neural crest origin, has been documented in cutaneous melanoma [17]. Elevated YB-1 expression is significantly upregulated in primary melanomas and is associated with worse survival outcomes. Experimental data suggests that YB-1 promotes melanoma progression and metastasis [17].

### 2.5. Microphthalmia Transcription Factor (MITF) and Transient Receptor Potential Melastatin-1 (TRPM1)

Microphthalmia transcription factor (MITF) plays a critical role in the development and function of melanocytes [18]. In melanoma, MITF protein functions as a transcription factor involved in proliferation and differentiation. However, MITF’s function in melanoma varies across specimens, where in advanced melanoma MITF is typically downregulated, but has been amplified in select cases [19]. Transient receptor potential melastatin-1 *(TRPM1)* is a calcium-permeable ion channel originally identified as a tumor suppressor gene and downregulated in metastatic melanoma [20]. It is transcriptionally regulated by MITF, and its decreased expression correlates with tumor aggressiveness [20,21].

#### Summary

Immunohistochemistry-based biomarkers including PRAME, Ki-67, AMBLor, YB-1, TRPM1, and MITF have been investigated as prognostic markers in cutaneous melanoma. Evidence is strongest for PRAME and Ki-67, with elevated expression linked to poorer OS and higher SLN positivity. AMBLor positivity has been associated with reduced recurrence risk in early-stage melanoma, while data for YB-1, TRPM1, and MITF continue to emerge. Currently, none of these biomarkers are included in NCCN or AJCC guidelines, though PRAME is increasingly adopted as a diagnostic adjunct.

## 3. Circulating Biomarkers and Liquid Biopsies

### 3.1. Lactate Dehydrogenase (LDH)

Lactate dehydrogenase (LDH) is a key metabolic enzyme upregulated in many cancers that contributes to tumor growth and metastatic progression. LDH is incorporated into the TNM (tumor/node/metastasis) staging of melanoma, where elevated expression is associated with worse prognosis [22]. In a study of 121 melanoma patients, LDH showed 92.2% specificity and 41.9% sensitivity for identifying regional or distant metastases (stage III–IV disease) [23].

Serum LDH is also an independent prognostic factor for survival and is included in the AJCC classification for stage IV melanoma [22,24]. LDH has also been evaluated as a biomarker for melanoma patients receiving anti-programmed death-1/programmed death-ligand 1 (PD-1/PD-L1) therapy, where elevated baseline LDH levels are associated with reduced OS (HR = 2.44; 95% CI, 1.95–3.04; *p* < 0.001) and shorter PFS (HR = 1.61; 95% CI, 1.34–1.92; *p* < 0.001) [25].

### 3.2. Serum S100 Calcium-Binding Protein B (S100B)

Serum S100 calcium-binding protein B (S100B) plays a key role in cytoskeletal integrity, regulating the cell cycle, and promoting apoptosis. It is one of the most studied biomarkers, released into the bloodstream by melanoma cells upon cellular damage or death [26]. Elevated S100B levels have been linked to worse outcomes in melanoma [27]. Notably, S100B is among the serum protein biomarkers with the most robust clinical evidence supporting its role in melanoma surveillance, particularly for monitoring recurrence during follow-up [26].

S100B levels correlate closely with tumor burden, with higher concentrations observed in advanced compared to early-stage disease [27]. A recent meta-analysis of melanoma follow-up studies found that S100B surpassed LDH in detecting recurrence, demonstrating a higher area under the curve (AUC) and greater sensitivity while maintaining high specificity [27].

Serum S100B has also shown utility as an early indicator of treatment response in melanoma patients receiving immunotherapy. In a study by Wagner et al., baseline S100B levels strongly correlated with overall survival in patients treated with anti–PD-1 agents [28].

Despite strong supporting data, S100B has not been incorporated into the AJCC staging system. LDH remains the only serum biomarker formally recognized. This reflects practical and historical considerations rather than biological inferiority. LDH is globally available, inexpensive, and standardized, whereas S100B has been regionally adopted and lacks widespread assay standardization, limiting its broader implementation [26].

### 3.3. Melanoma-Inhibiting Activity (MIA) Protein

Melanoma inhibitory activity (MIA) protein plays a role in melanoma progression by promoting metastasis and contributing to tumor-associated immunosuppression. It is considered a melanoma-specific biomarker because it is absent in normal melanocytes and benign melanocytic nevi but strongly expressed in malignant melanoma cells [29]. Elevated serum MIA levels have been associated with advanced disease stage, increased tumor burden, and worse overall prognosis [29].

In recent studies, MIA has also been evaluated as a dynamic marker for treatment monitoring, particularly in the context of *BRAF*-mutant melanoma [30]. In patients with BRAF V600 mutations undergoing targeted therapy with BRAF inhibitors (iBRAF), MIA levels, alongside S100B and LDH, were shown to significantly decline within the first month of treatment [30]. These reductions correlated with clinical response and PFS, suggesting that early decreases in MIA may serve as indicators of therapeutic efficacy.

Although MIA is less commonly used than S100B or LDH in clinical practice, it provides greater specificity for melanoma and may have added value when combined with other biomarkers. Further studies are needed to validate its role in routine surveillance and to determine its utility in predicting long-term outcomes across different therapeutic settings.

### 3.4. Liquid Biopsy Biomarkers (Cell Free DNA (cfDNA), Cell Free RNA (cfRNA), Circulating Tumor DNA (ctDNA))

Liquid biopsy approaches such as circulating cell-free DNA (cfDNA), cell-free RNA (cfRNA), and particularly circulating tumor DNA (ctDNA) are being investigated as prognostic and predictive biomarkers in melanoma.

ctDNA represents the fraction of cfDNA derived from tumor cells and is typically more fragmented than cfDNA of non-tumor origin. In practice, ctDNA is commonly measured by droplet digital PCR or next-generation sequencing (NGS), with results reported as mutant allele fraction (normal limit of detection ~0.1–0.5%) or copies/mL of plasma. It has emerged as a clinically relevant blood-based biomarker across multiple solid tumors, including melanoma [31,32]. In a multicenter study, ctDNA was detectable in 43.8% of patients with stage III melanoma, where its presence was significantly associated with recurrence at distant or multiple sites (*p* < 0.05) [33]. Palacios-Diaz et al. reported that ctDNA detection in plasma was significantly associated with disease progression (*p* = 0.011), overall mortality (*p* < 0.001), and melanoma-specific death (*p* < 0.001) [33]. Aoude et al. similarly demonstrated that patients with stage IV melanoma and low concentrations of ctDNA (<10 ng/mL) had better disease-specific survival and PFS [36]. Low ctDNA levels have demonstrated prognostic value in melanoma, correlating with improved distant metastasis-free survival (DMFS) during treatment and more favorable overall outcomes. Eroglu et al. reported that melanoma patients receiving adjuvant therapy who became ctDNA-positive, or remained persistently positive, had significantly worse DMFS compared with ctDNA-negative patients 3.5 months after surgery (HR = 34.54; *p* < 0.001) [37]. In sum, detectable ctDNA at baseline, increasing ctDNA levels, or reduced clearance of ctDNA during therapy correlate with worse outcomes. However, pre-analytic factors including time to plasma separation (>2 h) or the use of non-stabilizing blood tubes can diminish ctDNA yield.

Circulating cfDNA comprises extracellular DNA fragments released mainly from apoptotic and necrotic cells, but also through active processes such as neutrophil extracellular trap formation; erythroid precursors may contribute under certain conditions [32,34]. One study by Váraljai et al. analyzed cfDNA concentrations among patients with metastatic melanoma, compared to healthy controls, and reported that higher baseline cfDNA concentrations were significantly associated with higher AJCC stage, presence of metastases and shorter OS [35]. Cell-free RNA (cfRNA), particularly circulating microRNAs (miRNAs), is emerging as a promising non-invasive biomarker in melanoma by enabling pan-tumor monitoring and treatment response surveillance. Distinct plasma miRNA patterns can differentiate melanoma patients from healthy individuals with high sensitivity and specificity [35]. Specific circulating miRNAs have been shown to carry prognostic significance, with elevated *miR-221* correlating with thicker primary tumors, metastasis and worse survival outcomes. Additional specific messenger RNAs (mRNAs) such as *KPNA2*, *DTL*, *BACE2*, and *DTYMK* have been utilized as a panel for liquid biopsy in melanoma detection [38]. Low baseline cfRNA levels were associated with significantly longer PFS (*p* = 0.0349) and improved OS (*p* = 0.0393) [38]. Other miRNAs can reflect treatment response or recurrence likelihood. Taken together, cfDNA and cfRNA biomarkers show promise for enabling earlier detection of melanoma, informing prognosis and allowing dynamic, non-invasive monitoring of therapeutic response.

#### Summary

Among circulating biomarkers, LDH has the strongest evidence base and remains the only serum marker incorporated into AJCC staging for melanoma, supported by consistent findings from large multicenter studies. By comparison, S100B and MIA show moderate-quality evidence and in some studies may outperform LDH for recurrence detection, but their clinical adoption has been limited by lack of assay standardization and regional variability in use. Emerging liquid biopsy approaches, including ctDNA, cfDNA, and cfRNA, provide additional prognostic information, with ctDNA in particular associated with early relapse, shorter PFS, and worse OS. Notably, ctDNA has demonstrated prognostic value independent of AJCC stage.

## 4. MicroRNA

### 4.1. miR-200a-3p

*miR-200a-3p* has demonstrated both diagnostic and prognostic relevance. Prodan et al. reported that elevated circulating levels of *miR-200a-3p* were associated with reduced OS in patients with advanced-stage melanoma [39]. In addition, higher expression levels of *miR-200a-3p* were associated with increased risk of mortality (HR = 2.28; *p* = 0.001). In vulvar melanoma, *miR-200a-3p* expression was significantly lower than in primary cutaneous melanoma (*p* = 0.0008) [75].

### 4.2. miR-335-5p

Elevated circulating levels of *miR-335-5p*, have been linked to worse OS in melanoma, potentially through regulation of metastasis-associated pathways. In tumor tissue, *miR-335* expression is reduced relative to adjacent non-tumor tissue, yet within melanoma samples higher expression correlates with greater invasive depth, lymph node metastasis, and advanced stage, underscoring its prognostic significance [39,76]. Furthermore, patients with high expression levels had significantly shorter median survival times, compared to patients with low expression [39]. Collectively, these findings support *miR-335-5p* as a marker of poor prognosis in cutaneous melanoma.

### 4.3. miR-451a

In contrast to the aforementioned microRNAs, *miR-451a* is considered a favorable prognostic and diagnostic biomarker [39,77]. Higher expression of *miR-451a* was associated with a lower risk of a higher Breslow index [77]. *miR-451a* has also been found to correlate with patient responsiveness to anti-PD1 therapies in patients with advanced melanoma [78].

### 4.4. miR-29b-3p

Elevated levels of *miR-29b-3p* have also been associated with better survival outcomes and correlate with improved response to ICI therapy (OR = 2.35, 95% CI: 1.15–4.79) [39]. Prodan et al. showed downregulation (fold change = 0.92) of *miR-29b-3p* in melanoma [77]. Lower expression was also strongly associated with greater tumor thickness, with reduced *miR-29b-3p* linked to a higher Breslow index (OR = 2.51).

### 4.5. miR-424

Xu et al. reported that elevated *miR-424* expression was associated with more aggressive melanoma subtypes and correlated with reduced disease-free survival (DFS) and OS, indicating its role as a marker of poor prognosis [40]. Furthermore, *miR-424* expression was correlated with tumor thickness (*p* = 0.031), metastasis (*p* = 0.010), tumor stage (*p* = 0.005) and ulceration (*p* < 0.001) [40]. Du et al. supported these findings demonstrating that *miR-424-5p* was highly expressed in melanoma cells and their exosomes [79].

#### Summary

Evidence for miRNAs as melanoma biomarkers is emerging but remains limited. Elevated *miR-200a-3p, miR-335-5p,* and *miR-424* have been associated with worse OS, whereas higher levels of *miR-451a* and *miR-29b-3p* correlate with improved survival and enhanced response to ICIs. Most findings are based on single-center, retrospective studies that did not control for AJCC staging variables, making it unclear whether these associations are independent of established prognostic factors. At present, miRNAs remain investigational, and larger prospective studies are required to define their role in clinical decision-making.

## 5. Genomic and Transcriptomic

### 5.1. Gene Expression Profile (GEP) Analysis

Gene expression profiling (GEP) evaluates multiple mRNAs in melanoma tissue, using methods such as microarray analysis, mRNA sequencing, or reverse transcription PCR (RT-PCR), to predict metastasis and recurrence risk and potentially guide treatment and surveillance decisions [80,81].

11-Gene GEP: This qRT-PCR–based assay generates molecular scores for prognostic stratification [41]. In 245 patients with AJCC stage II cutaneous melanoma, high scores predicted significantly worse outcomes than low scores, including melanoma-specific survival (*p* = 0.018), DMFS (*p* = 0.005) and RFS (*p* = 0.009) [41]. The 11-GEP score remained an independent prognostic factor even when accounting for tumor thickness and age (HR = 1.21, and 1.05, respectively.) [41]. Despite prospective validation, clinical adoption is minimal due to limited cohort sizes and unclear impact on management decisions.

31-Gene GEP: Evaluated across 17 Surveillance, Epidemiology and End Results (SEER) registries (n = 4687), this assay stratifies patients into three risk categories: Class 1A was defined as low risk, class 1B/2A was intermediate risk, and class 2B was considered high risk. Bailey et al. found that class 2B patients experienced significantly worse OS (HR = 2.39), compared to Class 1A [81]. This model offers the largest real-world validation and broader clinical uptake but questions remain regarding its impact on clinical decision-making.

CP-GEP: This hybrid model integrates clinicopathologic features with gene expression to improve risk stratification in stage I/IIA melanoma [42,82]. It identifies high-risk patients for relapse while recognizing those with <5% SLN metastasis risk who may avoid biopsy [42,82,83]. Eggermont et al.’s model demonstrated high negative predictive value (90.5%, 95% CI: 77.9–96.2%) and superior relapse-free survival stratification (HR = 2.98; *p* < 0.0001) [42]. While this assay shows promise, it is still investigational and has limited clinical adoption.

#### Summary

GEP assays can provide risk stratification beyond traditional AJCC criteria. The 11-gene model shows prognostic value but has minimal uptake. The 31-gene model offers the largest validation cohort and broader adoption but unclear management impact. The CP-GEP model uniquely integrates molecular and clinical data for SLN decision-making but requires further validation. Key debates remain concerning clinical utility, cost-effectiveness, and which model best guides management decisions.

### 5.2. Telomerase Activation Through Telomerase Reverse Transcriptase (TERT) Promoter Mutation

Telomerase activation via telomerase reverse transcriptase (*TERT*) is a fundamental driver of carcinogenesis. In melanoma, *TERT* promoter mutations are associated with worse clinical outcomes. A meta-analysis of more than 2500 cases demonstrated that *TERT* promoter-mutations had significantly reduced OS compared with *TERT*-wild type (HR = 1.43; 95% CI, 1.05–1.95) [43]. The adverse prognostic effect was even stronger in melanoma-specific survival and in subgroup analyses of non-acral cutaneous melanoma [43]. The oncogenic role of *TERT* is further supported by findings in uveal melanoma, where activating promoter mutations have been identified in primary tumor samples, reinforcing its importance in melanoma pathogenesis through telomerase reactivation and cellular immortality [84].

### 5.3. Tumor Mutational Burden (TMB)

Tumor mutational burden (TMB) refers to the total number of somatic mutations within a tumor’s genome and has been shown to correlate with response to immunotherapy [85]. In 2022, Ning et al. published a meta-analysis evaluating the association between TMB and ICI outcomes in melanoma. They reported that high TMB was significantly associated with improved OS (HR = 0.49; 95% CI, 0.33–0.73; *p* = 0.001) and PFS (HR = 0.47; 95% CI, 0.33–0.68; *p* < 0.001) compared with low TMB [44]. These findings suggest that TMB may serve as a predictive biomarker for response to PD-1/PD-L1 and cytotoxic T-lymphocyte-associated protein 4 (CTLA-4) blockade, with higher TMB indicating a greater likelihood of benefit. Real-world data further support this association, showing that high TMB was independently predictive of progression-free survival and OS following first-line ICI therapy, reinforcing its potential role in guiding treatment decisions [45].

### 5.4. BRAF Mutation

The *BRAF* mutation, specifically the single point mutation V600E, is one of the most frequently mutated oncogenes in melanoma [46]. Pathogenic gene variants in the *BRAF* gene occur in about 50% of patients with melanoma [46]. This constitutively activating mutation drives tumor cell proliferation, survival, invasion, and metastasis [46]. Assessment of *BRAF* mutational status is now a standard component of the diagnostic workup for advanced melanoma [47]. *BRAF*-mutated melanomas are generally more aggressive in stage IV disease and are associated with reduced survival [86]. Moreover, patients with localized *BRAF*-mutant melanoma have demonstrated poorer DFS compared with those harboring wild-type tumors (HR = 2.2; 95% CI, 1.1–4.3) independent of AJCC staging [87].

### 5.5. Neuroblastoma RAS (NRAS) Mutation

*NRAS* mutations, present in approximately 15–25% of melanomas, define a subset of tumors that are generally more aggressive and associated with poorer outcomes compared to *NRAS* wild-type disease [48,49]. They are most frequently observed in nodular melanoma [49].

*NRAS* mutations have been linked to a higher frequency of nodal relapse (*p* = 0.013) and development of metastatic disease (*p* = 0.021) [48]. However, *NRAS* status does not appear to predict treatment efficacy, as studies have shown no significant differences in progression-free or OS among *NRAS*-mutant versus wild-type patients treated with anti–PD-1–based ICIs [50].

### 5.6. Cyclin-Dependent Kinase Inhibitor 2A (CDKN2A) Deletions/Mutations

Loss of the tumor suppressor gene, cyclin-dependent kinase inhibitor 2A (*CDKN2A*), is a frequent driver of melanoma progression. Inactivation may occur through somatic mutations, deletions, epigenetic alterations or inherited variants [51,52]. Patients with germline *CDKN2A* mutations tend to develop multiple primary melanomas (*p* < 0.001) and present, on average, 15 years earlier than those with sporadic disease (*p* < 0.001). OS and RFS do not appear significantly different between germline mutation carriers and non-carriers [53]. However, van Doorn et al. reported that familial melanoma patients with *CDKN2A* mutations had significantly worse melanoma-specific survival compared with wild-type cases, but this difference observed only in those not undergoing regular dermatologic surveillance [54].

### 5.7. Tissue Epigenetic Biomarkers (DNA Methylation)

DNA methylation is an important epigenetic mechanism with emerging utility as a biomarker in cutaneous melanoma. A 2024 study identified a three-CpG methylation signature significantly associated with OS in cutaneous melanoma, which was validated in two independent cohorts and remained prognostic across clinical subgroups [55]. Similarly, Huo et al. developed a novel prognostic biomarker based on relative methylation orderings of eight pairs of loci, stratifying patients into high- and low-risk groups [56]. The ‘low-risk’ (hypomethylated) group demonstrated greater CD8^+^ T-cell infiltration and higher tumor mutational burden (*p* < 0.05), correlating with improved treatment responses compared to the high-risk (hypermethylated) group [56]. Furthermore, global DNA methylation levels assessed by 5-methylcytosine staining were linked to melanoma aggressiveness in a recent study by Meevassana et al. [57]. Low 5-methylcytosine expression was significantly associated with greater Breslow thickness (comparing levels 2 and 4; *p* = 0.046), presence of ulceration (*p* = 0.039), and poorer OS (*p* = 0.027), suggesting that hypomethylation may be linked to more aggressive disease and worse outcomes [57].

### 5.8. Long Noncoding RNAs (lncRNA)

A 2024 meta-analysis by Masrour et al. reported that long noncoding RNAs (lncRNAs) may serve as prognostic biomarkers in melanoma, as altered lncRNA expression significantly associated with OS (HR = 2.72; *p* < 0.0001) [58]. Several lncRNA-based expression signatures have since been developed to improve risk stratification and correlate with clinical outcomes. Guo et al. identified immune- and ferroptosis-related lncRNA panels that independently predicted survival and reflected tumor immune and metastatic characteristics, suggesting that patients with higher expression of their five-gene lncRNA signature might benefit from ICI therapy [59]. Similarly, Zhou et al. (2021) developed a 15-lncRNA signature that predicted prolonged survival in advanced melanoma patients treated with anti–PD-1 immunotherapy [60]. However, because few lncRNAs appear consistently across studies, further validation is needed to establish the prognostic significance of individual lncRNAs.

#### Summary

Gene expression profile (GEP) assays provide additional prognostic information beyond Breslow thickness, ulceration and SLN status, particularly in patients with stage I–II melanoma where recurrence risk is often underestimated. The 11-gene model has demonstrated prognostic value but has seen limited uptake in clinical practice. By contrast, the CP-GEP model integrates gene expression with age and Breslow thickness to refine SLN biopsy decision-making, though broader validation is still required before incorporation into guidelines. Mutational biomarkers also offer important insights; TERT promoter mutations and CDKN2A alterations have been associated with aggressive tumor features and shorter recurrence-free duration, but standardized clinical integration remains lacking. Tumor mutational burden (TMB) has emerged as an investigational biomarker predicting immune checkpoint inhibitor (ICI) therapy responsiveness, with higher TMB linked to improved PFS and OS. Of the genomic/transcriptomic biomarkers discussed, only BRAF V600E/K mutations are routinely tested in practice, as they directly guide eligibility for BRAF ± MEK inhibitors independent of AJCC staging. NRAS mutations correlate with higher recurrence risk and poorer OS, though no targeted therapies are currently approved. Importantly, TERT and CDKN2A mutations have not consistently demonstrated independence from established AJCC prognostic factors.

Beyond genomic mutations and gene expression profiles, epigenetic biomarkers and long noncoding RNAs represent an emerging area of interest. Aberrant DNA methylation patterns at promoter regions and other epigenetic modifications have been associated with tumor progression, immune evasion, and adverse survival outcomes in melanoma. Similarly, dysregulation of lncRNAs has been linked to tumor invasiveness, metastatic potential, and resistance to systemic therapy. While these findings are promising, studies remain limited to small cohorts, and external validation is required before these biomarkers can influence clinical management.

## 6. Immune-Related Markers

### 6.1. Programmed Death-Ligand 1 (PD-L1)

Programmed death-ligand 1 (PD-L1) expression has been associated with improved outcomes in melanoma patients treated with anti-PDL-1 therapy such as pembrolizumab [88]. In advanced melanoma, higher response rates have been observed in patients with PD-L1–positive tumors, although thresholds for positivity (commonly ≥1% or ≥5% PD-L1–positive cells) vary across studies and assays, limiting comparability. PD-L1 expression, particularly when co-occurring with *BRAF* V600 mutations, has also been linked to longer overall survival following ICI therapy [61]. In a real-world analysis, Ellebaek et al. demonstrated that PD-L1 expression (threshold defined as >1%, assay clone and platform not specified) was an independent positive prognostic biomarker for melanoma-specific survival in patients receiving ICI therapy (HR = 0.66; 95% CI 0.52–0.83, *p* < 0.001) [62].

### 6.2. Lymphocyte Activation Gene-3 (LAG-3)

Lymphocyte activation gene-3 (LAG-3) is an inhibitory receptor expressed on T cells that has emerged as a potential predictive marker of responsiveness to ICI therapy, particularly PD-1 blockade. In one study of 11 melanoma patients who had completed anti–PD-1–based treatment, LAG-3 expression was detected in 81% of cases [64]. Responders to ICIs demonstrated significantly higher proportions of LAG-3^+^ cells compared with non-responders (*p* = 0.0210), and patients with ≥1% LAG-3^+^ tumor cells had significantly longer PFS than those with <1% LAG-3 (*p* = 0.0037) [64]. Similarly, Armura et al. identified LAG-3 as a prognostic biomarker, reporting that elevated LAG-3 expression correlated with poor prognosis in ocular melanoma (*p* < 0.0001) [65]. These apparently inconsistent findings likely reflect differences in melanoma subtype, treatment context, scoring methodology, and clinical endpoints. In cutaneous melanoma treated with anti–PD-1, LAG-3 positivity aligns with ICI responsiveness and longer PFS, whereas in ocular melanoma, high LAG-3 expression is instead associated with adverse outcomes.

### 6.3. T Cell Immunoglobulin and Mucin Domain-Containing Protein 3 (TIM-3)

T cell immunoglobulin and mucin domain-containing protein 3 (TIM-3) is an immune-checkpoint protein that has recently been investigated as a potential biomarker in melanoma. Machiraju et al. analyzed tumor samples from 90 patients with advanced cutaneous melanoma and found that increased filtration with TIM-3^+^ T cells in pretreatment metastases was associated with significantly shorter PFS under anti–PD-1 therapy (*p* = 0.019) [66]. In contrast, Wiecken et al. reported that high TIM-3 expression correlated with significantly longer overall OS in the anti-PD-1 treatment group (*p* = 0.007) [67]. TIM-3 has also been studied as a circulating biomarker: Navani et al. observed that TIM-3 expression on CD4^+^ T cells was significantly higher in non-responders compared to responders (27.8% vs. 13.4%, *p* = 0.01), suggesting it may serve as a marker of treatment failure [68]. As with LAG-3, these inconsistent findings likely reflect differences in where TIM-3 expression is measured (tumor tissue vs. circulating T cells), the treatment context, and the clinical endpoints analyzed. Overall, high TIM-3^+^ infiltration in pretreatment metastases has been linked to shorter PFS with anti–PD-1, whereas other cohorts report an association with longer OS.

### 6.4. Cytotoxic T-Lymphocyte Antigen-4 (CTLA-4)

Cytotoxic lymphocyte antigen-4 (CTLA-4) is an immune-checkpoint receptor that regulates T-cell activity and has been evaluated in the context of cutaneous melanoma. In a retrospective study of 432, germline variants of CTLA-4, such as the c.-1577 AA genotype, were identified as independent predictors of worsened event-free survival at 60 months (52.2% vs. 71.1%, *p* = 0.02 [69]. With the advent of anti–CTLA-4 therapies such as ipilimumab, attention has turned to CTLA-4 expression as a potential predictor of treatment response. Mastracci et al. and Pistillo et al. reported that high CTLA-4 expression on tumor-infiltrating lymphocytes and tumor cells was associated with improved clinical response to ipilimumab [70,71].

### 6.5. Melanocortin-1 Receptor (MC1R) Variants

Germline variants in the melanocortin-1 receptor (*MC1R*) gene, which affect pigmentation and UV susceptibility, have been studied in melanoma. In a large cohort, *MC1R* status was not associated with OS in general; however, male patients carrying an *MC1R* variant showed a significant survival disadvantage compared with those with wild-type *MC1R* [72]. Beyond germline associations, Su et al. reported a stepwise increase in MC1R protein expression across melanoma progression, with higher levels in deeper primary lesions (*p* < 0.0001) and in ulcerated tumors (*p* = 0.0008) [73]. Elevated MC1R protein expression correlated with worse OS in both primary (*p* = 0.0031) and metastatic melanoma (*p* = 0.0034) [73]. *MC1R* variants may also impact treatment outcomes: metastatic melanoma patients with concurrent *BRAF* V600 mutations and *MC1R* variants experienced lower response rates and shorter PFS on *BRAF* inhibitor therapy [89].

#### Summary

Immune-checkpoint biomarkers in melanoma show variable levels of evidence and prognostic relevance. Among them, PD-L1 has the most consistent data, with tumor proportion scores ≥1% predicting improved response to anti–PD-1 and anti–PD-L1 therapies across multiple large studies. PD-L1 retains predictive value independent of AJCC stage, and its immunohistochemistry is routinely used in clinical practice to guide treatment selection. By contrast, findings for LAG-3 and TIM-3 remain inconsistent, reflecting differences in tumor subtype, treatment context, and study design. Elevated CTLA-4 expression has been linked to enhanced immune responses, though survival correlations are mixed. MC1R germline variants increase melanoma susceptibility, but their prognostic utility in the context of ICI therapy is limited. Overall, PD-L1 is the only immune-checkpoint biomarker with established clinical value, while others remain investigational and require further validation.

## 7. Therapy Associated and Investigational Biomarkers

In addition to prognostic markers, several biomarkers in melanoma are tied to therapy selection, particularly in the context of targeted and immunotherapies. The most clinically established is the *BRAF* V600E/K mutation which serves as a prerequisite for *BRAF* and *MEK* inhibitor therapy. *BRAF* inhibitors are small molecule drugs that inhibit mutant *BRAF* kinase in the *MAPK* pathway, causing tumor regression in melanoma [90]. BRAF inhibitors may be given in conjunction with *MEK* inhibitors which improve and prolong responses of the therapy [91]. As summarized in Table 2, this approach exemplifies the utility of molecular biomarkers in guiding FDA-approved therapy decisions.

ICIs such as anti-PD-1 antibodies are broadly effective across melanoma subtypes regardless of *BRAF* mutational status and have substantially improved survival [90,92,93]. Additionally, anti-*CTLA-4* antibody was the first approved immune-checkpoint inhibitor in melanoma and may be used alone or in combination with anti-PD-1 antibodies [90,92,95]. In select subtypes such as acral and mucosal melanomas, KIT mutations can guide off-label use of KIT inhibitors [90,95,98]. In addition, while not required for treatment selection, PD-L1 expression and tumor mutational burden are under investigation as predictors of response to immunotherapy responsiveness [90]. Oncolytic virus therapy (T-VEC) also provides benefits for patients with injectable, unresectable lesions [90,92]. Together, these examples reflect the evolving landscape of biomarker-driven therapy in melanoma (Table 2).

Beyond these validated markers, several investigational biomarkers are under active clinical or translational study for their potential to improve prognostic stratification and predict treatment outcomes. These include immune-checkpoint molecules such as *LAG-3*, which may inform responsiveness to dual checkpoint blockade; ctDNA, which can indicate minimal residual disease or early relapse; and TILs, which correlate with ICI responsiveness [63,99,100,101,102]. Additional emerging areas include gut microbiota composition, which may modulate ICI efficacy; tumor gene expression signatures (e.g., IFN-γ or MYC/E2F pathways); and epigenomic or proteomic markers that reflect immune resistance or tumor aggression [63,100,103,104,105,106,107]. Table 3 highlights several of these investigational biomarkers, including immune-checkpoint molecules like LAG-3, ctDNA, and tumor gene expression profiles that may inform immunotherapy response. Although promising, none are yet validated for routine clinical use, and further prospective studies are required to establish their clinical utility.

## 8. Limitations and Future Directions

This review is limited by several methodological challenges. Many included studies were retrospective and single-center, creating heterogeneity in assay platforms and cut-off values that compromises cross-study comparability. Furthermore, endpoint definitions varied across studies, and many analyses failed to adequately adjust for established AJCC prognostic factors such as Breslow depth, ulceration, and sentinel lymph node status, potentially leading to overestimation or underestimation of biomarker effects. Publication bias favoring positive findings represents an additional concern.

Future research should prioritize prospective, multi-center studies employing standardized assay platforms and pre-specified cut-off values. Decision impact trials are critically needed to determine whether incorporating these biomarkers into existing AJCC staging frameworks would meaningfully alter clinical management and improve patient outcomes. Additionally, evaluation of external validity must address practical considerations, including cost-effectiveness, accessibility, and health equity, to ensure that promising biomarkers can be successfully translated into routine clinical practice across diverse healthcare settings.

## 9. Conclusions

In the last five years, there have been significant advances in molecular and clinical assays that have expanded the repertoire of prognostic biomarkers for malignant melanoma. Emerging literature surrounding tissue-based markers appears promising for better risk stratification in early-stage disease, while microRNAs and gene expression sequences may aid in prognostic assessment. ctDNA may be beneficial in determining relapse risk, though further studies are needed. Finally, immune-related markers may help predict tumor response to ICI therapy, which can dramatically improve OS in patients with advanced melanoma. These recent developments underscore the importance of biomarker-driven approaches to diagnosis and prognosis, aimed at enhancing personalized care and promoting better outcomes for patients with malignant melanoma.

## Figures and Tables

**Table 1:** Summary of Updated Diagnostic and Prognostic Biomarkers in Malignant Melanoma. Abbreviations: OS: overall survival, SLN: sentinel lymph node, PFS: progression free survival, DFS: disease-free survival, ICI: immune-checkpoint inhibitor.

Biomarker	Gene	Association	Independence from AJCC Covariates	Clinical Readiness
Immunohistochemical and Tissue-Based Prognostic Markers				
PRAME [10,11,12,13]	*PRAME*	Elevated PRAME protein expression is linked to worse overall survival (OS).	Unclear	Validated/used
Ki-67 [10,14,15]	*MKi67*	Elevated Ki67 predicts worse OS and higher sentinel lymph node (SLN) positivity.	Yes	Promising
AMBRA1 and loricin 1 [16]	*AMBRA1*/*LOR*	Elevated AMBL or was associated with low risk of recurrence.	Yes	Promising
Y-box binding protein 1 [17]	*YB-1*	Elevated YB-1 expression is associated with epithelial-to-mesenchymal transition and poor survival in melanoma.	Unclear	Investigational
Microphthalmia transcription factor [18]	*MITF*	Acts as a transcription factor in melanoma; high expression promotes proliferation; low expression is linked to invasion and therapy resistance.	Unclear	Investigational
Transient receptor potential melastatin-1 [19,20,21]	*TRPM1*	Downregulated in metastatic melanoma; loss of TRPM1 is associated with increased invasiveness and poor prognosis.	Unclear	Investigational
Circulating Biomarkers and Liquid Biopsies				
Lactate Dehydrogenase (LDH) [22,23,24,25]	*LDHA*/*LDHB*	Elevated LDH expression is linked with worse OS and shorter progression-free survival (PFS).	Yes	Validated/used
S100B [26,27,28]	*S100*	Elevated S100B is linked to worse outcomes and advanced stages.	Unclear	Validated/used
MIA [29,30]	*MIA*	Elevated MIA is linked with advanced disease stage, worse tumor burden, and worse overall prognosis.	Unclear	Validated/used
Circulating Tumor DNA [31,32,33,34,35]	*ctDNA*	Plasma ctDNA detection was associated with an overall worse prognosis and mortality. Patients negative for *BRAF* mutation-positive ctDNA also had better responses to the MEK inhibitor (trametinib) and the *BRAF* inhibitor (dabrafenib). Low ctDNA concentrations are associated with better disease-specific survival and PFS, as opposed to higher concentrations.	Yes	Promising
Cell free DNA (CfDNA) and Cell free RNA (CfRNA) [34,35,36,37,38]	–	CfDNA presence correlates with higher tumor burden and advanced stage. Elevated CfRNA is linked with shorter PFS.	No	Investigational/promising
*MicroRNA*				
miR-200a-3p [39]	*miR-200a-3p*	Elevated levels were significantly associated with poorer OS.	Unclear	Investigational
miR-335-5p [39]	*miR-335-5p*	Elevated levels were significantly associated with poorer OS.	Unclear	Investigational
miR-451a [39]	*miR-451a*	Elevated levels correlated with improved survival rates.	Unclear	Investigational
miR-29b-3p [39]	*miR-29b-3p*	Patients with increased expression showed better survival outcomes.	Unclear	Investigational
miR-424 [40]	*miR-424*	Elevated expression has shown decreased OS and DFS compared to those with low expression.	Unclear	Investigational
Genomic and Transcriptomic Biomarkers				
11-gene GEP [41]	–	“High-risk” GEP score had significant differences in melanoma-specific survival, distant metastasis-free survival, and RFS compared to “low-risk”.	Unclear	Promising
CP-GEP [42] The CP-GEP model combines Breslow thickness and patient age, with the expression of eight genes in the primary tumor.	–	High-risk CP-GEP had considerably worse five-year RFS than the low-risk patient group.	Unclear	Promising
TERT Promoter Mutation [43]	*TERT*	*TERT*-mutated melanoma patients had a significantly worse OS.	Unclear	Promising
Tumor Mutational Burden (TMB) [44,45]	–	High TMB predicts OS after first-line ICIs and PFS.	Unclear	
BRAF [46,47]	*BRAF*	The *BRAF V600E* mutation promotes tumor proliferation and is associated with more aggressive disease, brain metastasis, and shorter survival in patients with advanced melanoma.	Yes	Validated/Used
NRAS [48,49,50]	*NRAS*	*NRAS* mutations are associated with aggressive tumor behavior, increased risk of nodal relapse and metastasis, and poorer outcomes.	Unclear	Promising
CDKN2A [51,52,53,54]	*CDKN2A*	Loss or mutation of *CDKN2A* is linked to earlier melanoma onset, multiple primary melanomas, and worse melanoma-specific survival in unscreened patients.	Unclear	Validated/used
Tissue Epigenetic Biomarkers (DNA methylation) [55,56,57]	–	Global hypomethylation and specific CpG signatures are associated with tumor aggressiveness and poor survival.	Unclear	Promising
Long noncoding RNAs (lncRNA) [58,59,60]	–	Altered lncRNA expression is significantly associated with OS; specific lncRNA panels may predict prognosis and ICI therapy response.	Unclear	Investigational
Immune-related Markers				
PD-L1 [61,62,63]	*CD274*	PD-L1 expression is correlated with improved OS.	Yes	Validated/used
LAG-3 [64,65]	*LAG3*	LAG-3 was associated with poor prognosis.	Unclear	Promising
TIM-3 [66,67,68]	*HAVCR2*	High TIM-3 protein expression on tumor-infiltrating lymphocytes is associated with resistance to anti–PD-1 therapy and variable survival outcomes.	Unclear	Promising
CTLA-4 [69,70,71]	*CTLA-4*	*CTLA-4* expression is associated with worsened event-free survival. Higher tumor/TIL expression may predict a better response to ipilimumab (anti-*CTLA-4*).	Unclear	Promising
MC1R Variants [72,73]	*MC1R*	Higher MC1R expression was associated with worse OS in primary and metastatic melanomas.	Unclear	Investigational

**Table 2:** FDA-approved Therapies for Malignant Melanoma and The Role of Associated Molecular or Genetic Biomarkers in Treatment Selection and Outcome Prediction. Abbreviations: OS: overall survival, ORR: overall response rate, PFS: progression-free survival).

FDA-Approved Therapy	Molecular/Genetic Biomarker	Role of Biomarker in Therapy Selection	Predictive Value for Treatment Outcome
BRAF inhibitors (vemurafenib, dabrafenib, encorafenib) [90,92,93,94]	BRAF V600E/K mutation	Required for therapy selection; only patients with BRAF V600 mutations are eligible	Strong predictor of response; high ORR and PFS improvement with BRAF/MEK inhibitors
MEK inhibitors (trametinib, cobimetinib, binimetinib) (used in combination with BRAF inhibitors) [90,92,93]	BRAF V600E/K mutation	Used only in combination with BRAF inhibitors in BRAF-mutant melanoma	Combination improves progression-free survival (PFS) and overall survival (OS) vs. BRAF inhibitor alone
Anti–PD-1 antibodies (pembrolizumab, nivolumab) [90,92,93,95,96,97]	None required; BRAF status not required	Used in all advanced melanoma regardless of mutation status	Predictive biomarkers (PD-L1, tumor mutational burden, TMB) not required for selection; benefit seen across subgroups
Anti–CTLA-4 antibody (ipilimumab) [90,92,95]	None required	Used in all advanced melanoma regardless of mutation status	No validated predictive biomarker; used alone or in combination with anti–PD-1
Oncolytic virus (Talimogene laherparepvec, T-VEC) [90,92]	None required	Used for injectable, unresectable cutaneous, subcutaneous, and nodal lesions	No validated predictive biomarker; best for limited disease burden
KIT inhibitors (imatinib, off-label use) [95,98]	KIT mutation (rare, acral/mucosal)	Considered in KIT-mutant melanoma (not FDA-approved for melanoma)	KIT mutation predicts response in select cases
PD-L1 inhibitors (atezolizumab, in combination) [90]	None required	Used in combination with BRAF/MEK inhibitors in BRAF-mutant melanoma	No validated predictive biomarker; PD-L1 expression not required

**Table 3:** Emerging Investigational Biomarkers Under Study for Prognostic and Predictive Use in Malignant Melanoma. Abbreviations: ICI: immune-checkpoint inhibitor).

Biomarker/Signature	Investigational Status	Potential Clinical Impact
LAG-3 expression [63,100]	Early-phase clinical/translational studies; not standard	May predict response/resistance to immune-checkpoint inhibitors (ICIs)
Circulating tumor DNA (ctDNA) [63,99]	Prospective validation ongoing; translational research	Early detection of relapse; dynamic monitoring of treatment response
Immune cell phenotyping (TILs, peripheral blood) [63,102]	Under investigation with single-cell and spatial profiling	May stratify prognosis and predict ICI response; not yet validated
Gut microbiota composition [63,100]	Exploratory clinical studies	Potentially modulates ICI efficacy; not yet actionable
Gene expression signatures (e.g., allograft rejection, IFN-γ, inflammatory response, E2F/MYC downregulation) [99,103,104]	Identified in transcriptomic studies; validation required	May predict benefit from ICI or targeted therapy; not yet standard
Mast cell and dendritic cell activation [107]	Multi-omics and translational studies	Negative correlation with ICI response; potential for risk stratification
Oncogenic pathway enrichment (JAK-STAT, RAS, MAPK, HIF-1, PI3K-Akt, VEGF) [107]	Multi-omics studies; not in clinical use	May predict ICI response or resistance; further validation needed
MicroRNA and protein expression profiles [105,107]	Identified in omics studies; not validated	Potential predictive/prognostic markers for ICI response
Intratumor heterogeneity (ITH) [103,107]	Genomic/transcriptomic studies	May predict resistance to immunotherapy; not yet clinically actionable
Epigenomic signatures (e.g., DNA methylation, chromatin modifiers) [100,106]	Early translational research	May influence ICI response; under investigation
Tumor-associated antibodies [102]	Single-cell and serological studies	Potential for non-invasive prediction of response; not validated

## Data Availability

No new data were created or analyzed in this study.
